# Pathology

**Published:** 1825-10-01

**Authors:** 


### PATHOLOGY.

Actio læsa suum designat singula morbum;

Præterea morbum partis mutatio signat.

---

1. *Hospital Reports, from l'Hotel Dieu.*† Of the acute diseases during the first quarter of the present year, about a fifth proved fatal, which is a large proportion of deaths, even in the midst of a bad epidemic. Of the chronic diseases, the rate of mortality was rather more

---

* Among other curtailments in the Periscope of this Number, and for the reasons already stated, we have been obliged to omit the Section on *Physiology*, as of less practical importance than some other sections.—*Ed.*

† Revue Medicale. Professor Recamier—M. Martinet. Tableau des Maladies Observées à l'Hotel Dieu pendant le Premier Trimestre de 1823. —*See the former Reports, p* 217 *of last Number.*

We hope and trust that the medical officers of our Metropolitan hospitals will follow the example of their Parisian brethren, in selecting intelthan one in four. The inflammatory complaints were chiefly of the digestive tube and of the lungs.

1. *Fever.* Two cases of fever, in this quarter, afforded proofs, if, indeed, proofs be still necessary, that the symptoms during life cannot always be connected with appreciable lesions in the dead body. One of the subjects was a young man, 18 years of age, who had had fever fifteen days before he entered the hospital. He then presented symptoms of slight pulmonary affection, to which were added, a few days afterwards, cephalalgia, inclination to vomit, yellow coat on the tongue, wandering pains in the abdomen, &c. In a day or two these subsided, but still the fever continued. There arising some tenderness of the epigastrium, twenty leeches were applied there, no antiphlogistic measures having been previously put in force. On the 20th February, being then able to get up a little every day, he was seized with spasms, which soon went off, leaving him pale and depressed in spirits during the remainder of the day. 21st. He complained of oppression about the chest, and extreme malaise—he had diarrhœa—the base of the thorax was the seat of acute pain—pulse small and concentrated. He got up to the close-stool, and there he suddenly and unexpectedly expired.

*Dissection.* The most rigorous examination could discover no trace of disease, to account for this sudden death, in any part of the body.

*Case* 2. Michelot, aged 19 years, of strong constitution, had been ill some days before he entered the Hotel Dieu, leeches having been applied to the head, and to the epigastrium. He was affected with diarrhœa and fever. Eighteen leeches more to the head, as he evinced some delirium. Next day, (7th February) the belly was rather swelled and painful. He was leeched there, and bled from the arm. The blood was inflamed. 8th. No amendment. The fever is higher—the diarrhœa continues. 9th. Much delirium—dilatation of the pupils—tongue dry. Twenty-five leeches behind the ears. 10th. In the same state. 11th and 12th. The intellects much clearer—the fever persists, but is moderate—the diarrhœa continues. 13th. There is some subsultus tendinum to day. 14th. Continues to sink. 15th. Died.

*Dissection.* The membranes of the brain were transparent, and the substance sound. No effusion in any part of the head. The thoracic viscera were perfectly healthy. *Abdomen.* The mucous membrane of the stomach and small intestines quite pale, and in the most perfect state of integrity—"dans le meilleur état possible"—the internal surface of the sigmoid flexure of the colon presented some very trifling blush of redness—all the other abdominal and pelvic organs remarkably sound.

---

ligent pupils to record the most interesting cases in a book, from whence reports might be made in some of the Medical Journals, from time to time, in an authentic shape. If they do not adopt some such plan as this, their reputations are likely to suffer from the garbled and falsified pictures which are presented of their practice by unauthorized reporters.

Well may the physician say that "certes, il est impossible de trouver ici une gastro-enterite, ou de reconnaitre les traces d'une arachnites." Let the disciples of Broussais and Clutterbuck ponder on these facts—and still aver that there is no such thing as idiopathic fever—that all fevers are gastro-enterites or phrenites!

*Diseases of the Encephalon.* We shall only notice one or two of these. The arachnitis was confined to the inferior surface of the cerebellum, with the formation of false membranes on the left lobe of this organ, and on the corresponding side of the tuber annulare. In the middle of the disorders occasioned by this state of things, giving rise to opisthotonos, strabismus, &c. the intellectual faculties remained unaffected, and the patient answered questions, and described his feelings to the last. This freedom of the intellect accorded perfectly with the lesions found on dissection. The arachnoid covering the parts abovementioned was alone inflamed, and that which enveloped the hemispheres was perfectly sound. We know that delirium never manifests itself when the cerebellic arachnoid *only* is inflamed.

*Case.* Lefevre, aged 54 years, having experienced severe reverses of fortune, was seized, on the 4th November, 1824, immediately after a moderate supper, with giddiness, weakness of the lower extremities, embarrassment of speech, drawing of the mouth to one side, and slight confusion of ideas. He was bled immediately, which relieved the symptoms, and in three or four days they entirely subsided. Early in February, 1825, he experienced new misfortunes, which were followed by such sensorial disturbance that he could neither read nor write—and soon afterwards he lost the power of clothing his ideas in appropriate language. Numerous leeches were applied to the head, but without good effect. On the 17th he entered the hospital. He now presented the following phenomena:—muscular power unimpaired—the senses and sensibility unaffected—the general health apparently good—the power of language very imperfect, he making use of words that were quite unintelligible or inapplicable to the questions asked. It is curious enough, that, although he could not answer questions in words, he could write down the most distinct and sensible answers. He could not read, or at least pronounce, the words which he had written. This man was ultimately discharged from the hospital without any amendment.

*Diseases of the Chest.* Our author observes, that the hospital presented to the students this quarter a disease which has hitherto passed undescribed by authors—namely, inflammation of the ultimate ramifications of the bronchia. This disease, he thinks, has been confounded with catarrhal inflammation of the larger air-tubes, with pneumonia, asthma, &c. though it greatly differs from them. Andral is the only author who has noticed, but in a very cursory manner, this disease. When the ultimate bronchial ramifications are inflamed, the chemical changes produced by respiration on the blood are incomplete—hence the deranged functions of respiration and circulation. The dyspnœa is exasperated by fits, and then becomes extreme. The pulsations of the heart are increased in frequency, corresponding with the acuteness and sensibility of the malady. Finally, the patient is thrown into a state of real asphyxia which soon proves fatal, the inflammation of the air-cells preventing the oxygenation or decarbonization of the blood. In the mean time, the chest emits its proper sound, the lungs being permeable to the air. This species of bronchitis merits, Recamier thinks, to be studied with more care than has hitherto been done, on account of its severity, and, indeed, fatality. A memoir on the subject is forthcoming, in a succeeding No. of the Révue Medicale. Three patients, affected with this disease, died in a few days from the commencement of the attack. In three others, the most active antiphlogistic and revulsive treatment saved life, though with much difficulty.

Our author observes, that the mortality from pulmonic inflammation was very moderate this quarter, being only five in thirty, or *one in six.* Now we really consider this a very high ratio of death from thoracic inflammation—an inflammation which we consider amongst the most manageable of all the internal phlegmasiæ.

Of the five who died of pulmonic inflammation, only one merited the notice of the reporter. It was a case where there was a coincidence between latent and evident pleuropneumony. The patient had been bled from the arm three times, and had a number of leeches applied, together with cupping-glasses. By these means, the fever had nearly been dissipated, and he was becoming convalescent, when he one morning presented a slight degree of dyspnœa, together with pain in the right side. Leeches were applied, but he suddenly expired the next day, to the astonishment of his medical attendants.

On examination, the left lung which had been inflamed was restored nearly to a state of integrity. The right lung, on the other hand, was hepatized, and the pleura diaphragmatica covered with thick false membranes.

During the whole of 1824 they did not meet with a single case of pericarditis. But three cases of this dangerous and insidious disease presented themselves in the course of two months, in the present quarter. One of these was affected with pleurisy in the left side, and also gastritis. These diseases were easily recognized, and treated accordingly; yet the patient died on the 7th day. On dissection, there was found, in addition to the diseases specified, a very extensive pericarditis, with false membranes on the surface, and flocculent effusion into the cavity of the pericardium.

It was not (as is often the case) till after the *post-mortem* appearances were noticed, that the physicians began to connect certain phenomena during life with the organic lesions found after death. Thus, it was remembered that the patient evinced a constant agitation and degree of malaise disproportioned to the other phlegmasiæ with which he was affected. The appearance of the countenance was also very striking, and the sensorial functions were more disturbed than in ordinary pleuritic and gastric inflammations.

*Abdominal Inflammations.* This section of our author's report contains but little that needs notice. We shall present the particulars of one case.

*Case.* Franhi Boile, aged 29 years, had been ill about a fortnight before he entered the hospital, his principal complaints being pain in the belly, and diarrhœa. When he was received, his face was flushed—there was some stupor—mouth and tongue dry, the latter red—abdomen swelled and painful on pressure—diarrhœa troublesome—temperature not greatly increased—pulse 93—sensible when spoken to—the thorax sonorous in every part, except under the right shoulder blade—breathing free. These symptoms increased for a few days, and then decreased. On the 8th day from his entering the hospital, there were symptoms of convalescence, and the patient began to take some food. On the 10th day, a small ecchymosis was seen on the left foot, which spread rapidly, and sloughed. The fever now entirely disappeared, and the patient became completely convalescent. Tonics and nourishing food were allowed. The wound having put on a good appearance, the diarrhœa and fever returned on the 33rd day, and in a few days more carried him off.

*Dissection.* The intestines were distended with fetid gas—and covered externally with a thin layer of whitish-looking matter, some spoonfuls of which could be collected in the pelvis. The vessels of the peritoneum were not at all injected. The mucous membrane of the stomach was pale, soft, and easily separated from the contiguous coat. The small intestines were sound throughout, except at the cœcal extremity, where two or three small ulcerations of the mucous membrane were seen. There were numerous ulcerations of the colon. There was no other morbid appearance in the abdominal viscera. The heart was flaccid, and its muscular substance very soft—the lungs were crepitous—the mucous membrane of the aërial passages of a violet colour, and covered with a frothy mucus.

*Colica Pictonum.* Of six cases, three were treated by leeching the abdomen, with the most decided advantage. One of these had undergone the treatment of La Charité, without any amendment. The symptoms were dissipated, like as by a charm, on the application of fifty leeches to the abdomen. This took place, too, in the case which appeared least of all inflammatory. In three other cases, the treatment was by narcotics and purgatives combined. One of these patients had a relapse, and returned to the hospital. Acupuncture was practised. The patient died 24 hours afterwards, and dissection could not detect any lesion to account for the death of the patient. These are the principal facts of any interest, which we have been able to extract from M. M. Recamier's and Martinet's reports for the first quarter of the present year.

2. *Disease of the Heart.** A female, aged 47 years, was seen by Dr. B. on the 25th January, 1825, complaining of some difficulty of breathing, and great sense of commotion at the heart and down the left side. She had been thus affected for 23 days, and attributed her complaint to cold and damp, after being heated with exercise. She had enjoyed good health, but had suffered considerable domestic anxiety, in consequence of the cares of a large family. *Present state.* Sleep disturbed—pulse 152, irregular and intermittent—heart beating with great violence, and not in consonance with the pulse at the wrist. By aid of the stethoscope, the want of regular alternation in the action of the auricles and ventricles could be perceived. The respiration was hurried, as after running. Bled from the arm to the extent of eight ounces, and an aperient mixture prescribed. 26th. No alteration for the better. The pulse could not be counted at the wrist. She was again bled to 15 ounces, by which she was relieved for a time. *February* 9. The difficulty of breathing was now great—her nights were sleepless—pulse still irregular—bowels costive. On examination with the stethoscope there was much mucous rattling (râle muqueux) at the upper part of the right lung, and a little in the left. Blister to the chest—aperient medicines. The action of the heart became more moderate, and the respiration easier. *February* 17. All the symptoms worse—anasarca of the legs in the day time. She lingered out till the 3d March, when she died.

*Dissection.* The right lung appeared healthy at its upper part, but the middle and lower lobes contained dark portions, with defined edges, which, when cut into, were solid. "*This alteration of structure appeared to have been produced by hæmorrhage into the substance of the lung which had caused ecchymosis around the ruptured vessel, rendering that portion solid, and thereby obliterating the air-cells.*" "A large portion of the lower lobe was also hepatized." There was about a pint and a half of serous fluid in the right cavity of the chest, with a few flakes of coagulable lymph. There was some inflammation in the pleura, especially where it is reflected over the diaphragm. In the left side there were about six ounces of water. In the lung of this side, there were some portions in the same state of disease as on the other side. The cavity of the pericardium contained about an ounce of serum. The heart was large, and both auricles and ventricles were distended with black blood. The parietes of the left ventricle were hypertrophied. The right auricle was considerably dilated. The tricuspid valve was thickened and cartilaginous—and the mitral valve was observed to be in a similar state. The semi-lunar valves of the aorta were diseased.

"The disease existing in the lungs," says Dr. Baker, "and that in the heart cannot, in my opinion, be regarded as holding any relation to each other, as cause and effect." Dr. Baker observes that the defect in the tricuspid valve would lessen the impetus of blood on the lungs, by

* Dr. Baker, Med. Phys. Journal, June 1825.

allowing a portion of that fluid to regurgitate into the right auricle. But he takes no notice of the defect in the mitral valve, which would cause a regurgitation into the left auricle, at each ventricular contraction, and thus keep the venous system of the lungs constantly gorged. We beg to differ in opinion, therefore, from Dr. Baker, and express our belief that the valvular disease of the heart did stand in the relation of cause to the hepatization in the lungs, and to the serous effusion in the cavities of the pleura. These effects were, no doubt, greatly increased or accelerated by the exposure to cold and wet; but still, the probability is, that they would not have taken place at all, had it not been for the cardiac disease previously existing.

---

3. *State of the Stomach in Phthisis.** It very generally happens that, when any one vital organ of the body is seriously affected, one or more other organs or structures become deranged, whether from sympathy, contiguity, or association of function. These secondary affections are too much neglected or overlooked by physicians, being considered as solely dependent on the primary malady, and not to be removed but through the medium of its cause. This is a mistaken notion, as attention to the secondary complaint will often signally mitigate the primary. At all events, a neglect of the former will very generally aggravate the latter. The stomach is frequently deranged in function and even in structure, this consecutive way—and especially in tubercular disease of the lungs. The object of M. Andral's paper is to demonstrate this: and not only this, but also to prove that, in the great majority of such consecutive affections of the stomach, the disease is a real phlegmasia of the chronic kind.

The frequency of gastric affections among the consumptive may be proved by the symptoms during life, and the *post mortem* examinations. During a period of five years' observation, under Lerminier, at La Charité, our author found that three fifths of those who died of phthisis pulmonalis presented unequivocal proofs of disease of stomach. The lesions observed were as follow:—1mo. In a considerable number of instances, a vivid injection of the mucous membrane, especially about the great curvature, without any manifest alteration in the thickness or consistence of the membrane itself, and without any derangement of the subjacent tissues. This injection was confined to the capillary system, and was clearly an inflammation, and not a mechanical stasis of the blood, a cadaveric effect. 2ndo. A brownish tint of the membrane, generally with thickening of its substance and some degree of hardening. 3tio. Most frequently of all, a softening of the mucous membrane of the stomach, in various degrees, sometimes accompanied by redness, sometimes by paleness of the part. 4to. Ulceration, but this very rarely. 5to. Still more rarely an alteration of texture in the subjacent coats of the stomach, consisting of thickening, induration, or tuberculation.

---

* M. Andral, Jun. Revue Medicale, Avril, 1825.

Of these various changes, and that the most frequently observed, there is but one which can be doubted as to its inflammatory origin :— this is the *softening* of the mucous membrane—so well described by modern pathologists, especially by M. Louis. One of the most remarkable instances of this kind which our author has met with was the following:

*Case.* A man, aged 35 years, died in La Charité, in the course of the month of June, 1824. During the three months which he spent in the hospital before his death, he never vomited any thing; but he complained of entire want of appetite, accompanied by an habitual sense of tightness about the epigastrium, changing into actual pain on taking any solid food or even drink. Wine produced a burning sensation accompanied by nausea. On opening the body, after death, the mucous membrane of the stomach was found, as it were, in a state of *debris*—the subjacent cellular tissue appearing naked, or nearly so, from the cardia to the pylorus, being of a white colour and somewhat thickened. In some places there were small remaining patches of the mucous membrane, like little islands scattered about. There were ulcerations in the intestines—the lungs tuberculous.

It was among phthisical patients that M. Louis generally found this softened state of the gastric mucous membrane. But, asks our author, is this softening a proof of gastritis?—From an examination of the anatomical characters, and from the symptoms during life, as well as from the analogous effects of inflammation on many other structures, our author comes to the conclusion, that this appearance in the inner membrane of the stomach in phthisis pulmonalis, is the product of phlegmasia.

That this softening results from an inflammatory process, he thinks is also proved by the effects of irritants, as poisons, for example, on the stomach. In examining the stomachs of drunkards who have died at La Charité, this softening of the mucous membrane was one of the most common appearances which our author met with. M. Cruveilkeir observed a gelatinous appearance in the stomachs of children who have died after being crammed with too much and indigestible food. It is well known that inflammation has the effect of softening the cerebral mass also. One reason more may be advanced on this side of the question—namely, that in cases where dissection shewed this softening of the stomach, the effect of tonics and stimulants, before death, was always injurious, while antiphlogistics invariably mitigated the symptoms.

From all these considerations, then, our author thinks himself authorised to conclude that more than one half of those who die of tubercular consumption in our hospitals, are also affected with chronic or acute gastritis. If this be the truth, or any thing near the truth, it should operate as a caution against a too precipitate use of tonics in phthisis—a mode of treatment which seems now to be coming into fashion.

4. *Intestinal Calculus.** A boy had been sickly from his infancy—his complaint being a purging and pain in his belly. The boy had passed his eleventh year, when Mr. Torbet was consulted. He was surprised to learn that this purging had continued for years back—the boy, during that time, having been obliged to be up two or three times in the night, and sometimes as often as every quarter of an hour, the stools being always thin and watery, sometimes yellowish, sometimes whitish. He ate little and was always thirsty. He had occasionally pain in his belly, where there was sometimes felt a hard lump. The boy was stunted in his growth, emaciated, and had a peculiar sharpness of expression in his countenance. In the right hypochondrium there was a perceptible fulness and hardness. Mr. T. was persuaded there was diseased liver or mesenteric glands, but delivered a guarded prognosis. *Light mercurials—frictions—flannels—leeches when there was acute pain.* Several months passed away without any amendment. In February, 1825, the boy was reported to be worse. Early in March our author saw him, and found much increase of emaciation—burning pain at the pit of the stomach—vomiting—great thirst. The lump was now very tangible in the right hypochondrium. Mr. T. again fortified himself in the belief that the liver was the organ affected.—He died in an hour after this interview.

*Dissection.* On opening the abdomen it was evident that the prognosis was wrong. The liver was sound. The ascending and transverse portions of the colon contained three concretions of unusual size, and weighing 12½ ounces. There was no thickening of the coats of the gut in the place where these calculi were lodged.

*Observations.* Our author appears to lay down a plan of symptoms diagnostic of "cases of this nature," from those present in the case narrated. But surely this is generalising on a very narrow foundation. The symptoms will vary much according to the portion of colon in which the concretion is lodged. As to Dr. Munro's proposal of cutting out these calculi, we can say nothing. The idea of getting into the cavity of the colon in that very limited space behind, where it is not covered by peritoneum, appears to us very chimerical. We agree with Mr. Torbet in thinking that there would be much less danger and difficulty in cutting through the abdominal parietes as near to the tumour as possible.

We cannot bring ourselves to believe that Mr. Torbet had good reason for his diagnosis. From the symptoms described, we certainly should not have concluded that the liver was the seat of the disease. An enlargement and induration of the liver, in such a tangible shape as was here presented, would have been accompanied by a very different train of symptoms, both positive and negative. The *paroxysms* of acute pain alone would have led us to suspect that some viscus was concerned where there were muscular fibres—to wit, the intestinal canal. This

* Mr. Torbet, Surgeon, Paisley. Ed. Journ. July, 1825. Dr. Kennedy, Med. Chir. Journal, September, 1817.

was a *positive* symptom. The absence of all icteritious and dropsical phenomena, was no unimportant *negative* indication. But we will say no more. Mr. Torbet will not make another mistake of the same kind, now that his attention has been excited to the diagnosis, by a very pardonable failure in this most difficult branch of our art.

Dr. Duncan has added a note on the nature of the concretions found in this case. It appears reasonable to suppose, that the *debris* of certain articles of vegetable food form the basis of these concretions, the earthy constituent being a kind of crystaline deposit from the intestinal fluids upon these nuclei. Dr. Duncan thinks that an attempt to dissolve these calculi is not unworthy of consideration. From the great solubility of the earthy phosphate of which they are chiefly composed, considerable impression might probably be made on them by the mineral acids, taken by the mouth or thrown up per anum.—"The use of *oats* and oatmeal in every shape should be positively interdicted." This prohibition of *oats* may sound a little strange in the ears of John Bull; but it is sufficiently significant in

"The land of mountain and of flood."

Before quitting this subject we beg to refer the reader to a very able paper on intestinal concretions, in the Medico-Chirurgical (monthly) Journal for September, 1817, by Dr. James Kennedy, of Glasgow. In that paper, Dr. K. relates the case of a female, aged 60 years, who, for a considerable time had experienced much torpor of the bowels, with difficulty and pain in evacuating them, requiring the constant use of laxatives. In the month of May, 1816, the constipation of bowels assumed an uncommonly obstinate form, and, for some days, Dr. Kennedy's patient was harrassed with severe and frequent paroxysms of tenesmus. While straining violently in the act of evacuating the bowels, she felt an unnatural sensation within the orifice of the rectum, and hence was induced to make an examination with her fingers. She felt a hard body completely obstructing the gut, and after much difficulty, and with the assistance of another female, effected its removal. The concretion was examined by Dr. Ure. It was an inch in its largest diameter, and three quarters of an inch in the smaller. In hardness, it was about equal to that of wax, but without its tenacity. At one extremity it exhibited concentric laminæ. Its sides had the lustre and marbled appearance of Castile soap. Its internal structure was granular, approaching to crystaline, with radiations from the centre to the circumference, of brownish and bright yellow lines, possessed of pearly lustre. Its weight was 167 grains. Its odour was strong, but by no means disagreeable. It was decidedly like that of musk, or more precisely that of ambergrease. But for various interesting details respecting the chemical analysis of this curious concretion, we must refer to the Journal before-mentioned, where the reader will find a considerable body of information on the subject in general, collected with much labour, research, and discrimination by Dr. Kennedy.

5. *Hydrophobia—extensive Neuritis.** Cases of hydrophobia are becoming more common, but not less fatal. The one which we are about to notice will not increase our hopes as to the efficacy of medicine in this dreadful disease. From two dissections of hydrophobic patients, at which we assisted, we have long been convinced that the pathologic condition existing when the symptoms actually appear is beyond the reach of art. Prevention, then, is our only chance.

In the January number of the Edinburgh Journal, Dr. Brandreth relates the case of a young man who died of hydrophobia, nearly 11 months after the bite. On the day of this young man's death, a rabid animal bit three persons, one of whom forms the melancholy subject of the present history. The patient was an artist of distinguished reputation. He received several lacerated and punctured wounds from the dog's teeth, in both wrists and in one hand, while rescuing his brother from the rabid animal. The most careful excision was practised within half an hour of the bite; "but as the worst wound was above the pisiform bone, and among the ligaments, it was difficult to do it effectually." He was desirous of having the arm amputated—and it was unfortunate (we impute no blame to the attendants) that his wishes, on this occasion, were not complied with. In hydrophobic excisions nothing is done while aught remains undone. Caustic was afterwards applied. This event happened on the 18th of October, 1824, and on the 12th January, 1825, symptoms of hydrophobia presented themselves. We need not here repeat details which are familiar to every one's ear. This unfortunate gentleman died on the third day—the only singularity in his case being the power, exerted by a terrible effort of the will, to swallow liquids almost to the last. Opium and colchicum were the principal medicines employed.

*Dissection.*—The dura mater was preternaturally vascular. The arachnoid was opake, and between it and the pia mater there was some serous effusion. "The pia mater was vascularity itself, and presented a complete mass of highly injected blood-vessels, the colour of which was not that of congestion, so much as of inflammation, approaching to scarlet." The medullary structure of the brain presented, when sliced, a number of bloody points. There was nothing particular in the lateral ventricles, except that the plexus choroides were very large, and of a dark reddish-brown colour. The cerebellum presented nothing remarkable, excepting a high degree of vascularity like the cerebrum. The vessels of the dura and pia mater were every where highly injected. The theca vertebralis was vascular; and, upon lowering the head, an ounce of serum ran from the sheath within the vertebral column.

*Neck and Arm.* The vasa vasorum of the carotid artery were rather tinged. The par vagum manifested a general blush of inflammation—the sheath of the nerve was injected with blood-vessels—and small ramifications of vessels were observed running parallel to the fibres in the cellular structure connecting the fibres. The 4th, 5th, 6th, and 7th

* Dr. Brandreth, Ed. Journal, January, 1825.

cervical nerves were next exposed, and the vascularity of their sheaths was strikingly evident, as well as the ramifications of blood-vessels, highly injected, between the fibres of each nerve. Some of them were so much altered in character as to resemble muscular fibre. Their external surface was almost scarlet, while the internal was of a pink colour, and the vessels could be distinctly seen by means of a small magnifying glass. The dissection was continued down the arm. The branches of the cutaneous nerves, upon the inner side of the arm, leading up from the cicatrix, which had been painful, presented the same appearance above described. The deep-seated nerves were not inflamed. The first absorbent gland, at the inner side of the elbow, and the absorbent glands leading to the axilla, together with the axillary glands, were examined, but presented no deviation from health. The cicatrix, which had been painful, was very vascular, "and this and another had a small portion of brown gelatinous substance effused in the cellular membrane under them." The others presented no such appearance.

The lining membrane of the mouth, underneath the tongue, was much inflamed. The fauces, uvula, and pharynx were in a high state of inflammation, particularly the pharynx. The œsophagus, especially near the cardia, was very vascular. The lining membrane was here almost completely denuded for the space of six or seven inches; what did remain peeled off like the cuticle of a blistered surface. Some patches of inflammation were seen on the internal surface of the stomach. The intestines were natural, but distended with air. The other viscera were healthy. The larynx and trachea were inflamed, as was the pleura. The lungs were in a state of extraordinary congestion.

*Spine.* "The bodies of the 3d, 4th, and 5th cervical *vertebræ* were raised from the forepart, and the *theca vertebralis* was found covered with injected blood-vessels. The *medulla spinalis* was natural; but at the point where the filaments pass from it to form a nerve, evident appearances of inflammation were visible. This was observed at the origin of all the cervical nerves in the portion of the medulla spinalis, which was exposed. Upon removing the bodies of the lumbar *vertebræ* in the same manner, the sheath was found extremely vascular, and there was some extravasated blood within it. The nerves in the pelvis, and those passing to the lower extremities, did not present the same inflammatory appearance as was observed in those of the neck and upper extremities." 237.

The above dissection, which lasted six hours, is very valuable, as being, perhaps, the most minute and careful of any recorded in this country. Great credit is due to the medical men who performed it. These were Mr. Bickersteth and Mr. Halton. (Do we recognise in the *former*, an old associate in anatomical pursuits, 25 years ago?) It holds out, as Dr. B. justly observes, no additional prospect of success in the treatment of hydrophobia. It can scarcely be doubted that the neuritis was here the chief actor in the fatal drama. We have little doubt that it is the *nervous system* which bears the onus in hydrophobia as well as in other animal poisons. But viewing the state of the nerves in this case, can we doubt that the inflammation had been going on for some considerable time before the hydrophobic symptoms broke forth ?—We think it must have been going on long before. Hence the small chance of curing hydrophobia when the explosion has once taken place. Complete excision then is the only safeguard—but where this cannot be done, or has been neglected, we confess that we should have most confidence in a pretty long continued course of mercury, kept up to the pitch of slight ptyalism. Such state makes a great revolution in the system, and is very incompatible with any constitutional disease. It is also highly calculated to repress or prevent inflammations, especially of the specific kind.

---

6. *Enlargement of the Heart.** A man, 62 years of age, came under Dr. Howison's care, on the 14th of January, 1823, complaining of uneasiness or dull pain at the lower part of the sternum, great occasional dyspnœa, especially when at stool, and aggravated at night, so that he is obliged to keep in the semi-recumbent position. He starts from his sleep, and has slight delirium at times. Countenance healthy, except a slight livor of the lips—lower extremities considerably œdematous—urine scanty and red—thirst—tongue foul—bowels costive—pulse full, and not above 30 in the minute, with irregularity. Reports that, for some months past, he has had severe palpitation of the heart. At present, it can merely be felt beating, and seems to labour in its function. He has enjoyed good health till within these three years. His complaint has rapidly increased since, by cold and fatigue, to which he had been subjected four months ago; says he has been much benefitted by digitalis, blisters, and aperients. We need not detail the treatment, which did no good. He got daily worse, and expired on the 19th of the same month.

*Dissection.* Lungs healthy—half a pint of water in the right side of the chest—about six ounces in the left side—pericardium appeared very vascular externally, looking as if it had been covered with powdered vermillion—no water in pericardio—*heart three times its natural size*, and very vascular, having also the vermillion-looking surface, with scattered spots, resembling purpura. The right chambers were greatly enlarged, and the margins of the valve thickened and cartilaginous. In the left side, the parietes of the ventricle were natural—the mitral valve cartilaginous. Nothing is said of the size of this cavity. The morbid appearances in the abdomen were not important. The liver, however, was enlarged, indurated, and of a pale colour.

Dr. Howison seems to think, that these morbid appearances do not account satisfactorily for the phenomena. We conceive that a heart enlarged to three times its natural size is no trifle. We have seen much less organic derangement than this produce all the symptoms above-mentioned (excepting, perhaps, the remarkable slowness of the pulse)

---

* Dr. Howison. Ed. Journal, July, 1825.

and destroy life. Diuretics are the most important remedies in such cases, with constant counter-irritation over the region of the heart. Life may often be considerably prolonged in such cases by judicious treatment.

